# Nephroprotective Effect of Herbal Extract* Eurycoma longifolia* on Paracetamol-Induced Nephrotoxicity in Rats

**DOI:** 10.1155/2019/4916519

**Published:** 2019-05-13

**Authors:** Sasikala M. Chinnappan, Annie George, Praveen Thaggikuppe, YogendraKumar Choudhary, Vandana K. Choudhary, Yesha Ramani, Rashmi Dewangan

**Affiliations:** ^1^Biotropics Malaysia Berhad, Shah Alam, Selangor, Malaysia; ^2^Etica Clinpharm Pvt. Ltd., Raipur 492001, Chhattisgarh, India

## Abstract

Paracetamol (PCM) is a well-known drug widely used for its analgesic and antipyretic properties. PCM is generally considered as safe but overdose of PCM can cause nephrotoxicity. Traditionally, herbs have been used for the treatment of drug or toxin-induced renal disorders and numerous medicinal plants were tested for nephroprotection effect in PCM-induced nephrotoxicity model. The aim of the present study was to evaluate the protective effect of the herbal extract* Eurycoma longifolia *(EL) against PCM-induced nephrotoxicity rat model. Forty Wistar rats were randomly divided into five groups of eight rats each: control (vehicle 10 ml/kg), PCM alone (200 mg/kg PCM), EL 100 (EL 100 mg/kg+200 mg/kg PCM), EL 200 (EL 200 mg/kg+200 mg/kg PCM), and EL 400 (EL 400 mg/kg+200 mg/kg PCM). All animals from control group received vehicle daily and animals from groups PCM alone, EL 100, EL 200, and EL 400 received repeated dose of PCM and the assigned treatment of EL daily for a period of 14 days. On the 15th day, serum creatinine, blood urea nitrogen, protein, and albumin were measured in blood and creatinine clearance was measured in urine collected over 24 hours. Kidney sections of all experimental groups underwent histopathological analysis. There was a significant (p<0.05) increase in serum creatinine and blood urea levels in the PCM alone group compared to the treatment groups due to nephrotoxicity. In the treatment groups, there was a dose-dependent protection against PCM-induced changes observed in serum total protein, albumin, urea, and creatinine. Significant (p<0.05) drop was seen in serum creatinine and blood urea content in EL 200 and EL 400 groups. Creatinine clearance significantly increased for EL 200 (p<0.01) and EL 400 (p < 0.001) groups. Serum total protein and serum albumin content were significantly increased (p<0.05) in EL 200 and EL 400 groups compared to PCM alone group. Histopathological examination (H&E staining) of the rat kidneys revealed severe degeneration in the PCM alone group, while there was evidence of significant dose-dependent protection in the treatment groups against PCM-induced changes. The serum and urine biochemical results and histopathology analysis of the kidney indicate the nephroprotective potential of EL extract against PCM-induced nephrotoxicity.

## 1. Introduction

Nephrotoxicity occurs when kidney-specific detoxification and excretion do not function optimally due to the damage or destruction of kidney function by exogenous or endogenous toxicants [1]. Drug-induced nephrotoxicity remains a major problem as use of nephrotoxic drugs is unavoidable in clinical setting. Paracetamol (PCM) is a commonly used drug, well-known for its analgesic and antipyretic properties [2]. Indeed, overdose of PCM in human is relatively common due to self-administration and is often associated with hepatic [3–5] and renal damage [6–9]. Even if nephrotoxicity is less common than hepatotoxicity in PCM overdose, renal tubular damage and acute renal failure can occur even in the absence of liver injury [10–12] and can be fatal in humans and experimental animals [13–15].To date, numbers of studies have been published to prove nephroprotective effect of medicinal plants by using PCM-induced nephrotoxicity in rats. Various PCM dose and modes of administration were used in these studies. In majority of the studies a single dose of PCM in the range of 400 mg/kg to 2000 mg/kg was administrated by oral or intraperitoneal (IP) route to induce toxicity [16–19]. Nephrotoxicity can also be induced by administering lower but repeated dose as suggested by some published work [20, 21]. A repeated daily dose of 200 mg/kg of PCM for 14 days by IP route was selected to induce nephrotoxicity in the present study as the selected dose was proven to cause nephrotoxicity [21].

Nephroprotective agents are material that has potential to minimize the effects of nephrotoxic agents. Medicinal plants have curative properties due to the presence of various complex chemical substances [22]. Ethnomedicinal plants from the traditional system of medicine viz Ayurveda and Unani, which are acclaimed by the Ayurvedic and Unani physicians to have nephroprotective properties and commonly used to treat various renal disorders, have been extensively investigated for their significant nephroprotective effects [23–25].

*Eurycoma longifolia *(EL) is a small Asian tree in the genus Eurycoma, commonly known as Tongkat ali or Long Jack, the roots of which are often called “Malaysian ginseng” [26]. It is used in the treatment of malaria, cancer and ulcers and for male sexual dysfunction and has been commonly prescribed in traditional medicine as a febrifuge and a remedy for dysentery, glandular swelling and fever [27]. It is popular as a singly or an essential component for the treatment of fevers, aches and sexual insufficiency as well as health supplements. EL has also been reported to have antioxidative properties due to its high concentration of superoxide dismutase [28].

The aim of this study was to investigate the nephroprotective properties of EL using a PCM-induced nephrotoxicity rat model. As there was no study or any preliminary work available on nephroprotection property of EL, a low 100 mg/kg (EL 100), medium 200 mg/kg (EL 200), and high 400 mg/kg (EL 400) doses were used in the study to investigate nephroprotective effect of EL. Acute or chronic PCM overdose is often associated with a wide range of metabolic disorders, including serum electrolytes, urea, and creatinine derangements. As such, elevations in the serum concentrations of these parameters, particularly, serum urea, and creatinine are considered reliable and well-documented parameters for investigating drug-induced nephrotoxicity in animals and humans [29–31]. The nephroprotective effects of EL extract in the PCM-induced nephrotoxicity rat model were evaluated by determining the levels of creatinine, blood urea nitrogen (BUN), protein, albumin, and creatinine clearance. Kidney sections were histopathologically analysed to assess ultra-structural changes in the rat kidney.

## 2. Materials and Methods

### 2.1. Collection and Preparation of EL Plant Extract

The EL extract used in this study was supplied by Biotropics Malaysia under the trade name of Physta®. It is a water extract of the roots of EL standardised based on specification of 0.8%-1.5% eurycomanone, not less than 22% of total protein, not less than 30.0% of total polysaccharide, and not less than 40.0% of Glycosaponin.

### 2.2. Preparation of PCM

Paracetamol and all other chemicals used were of analytical grade and purchased from M/s. Erba Mannheim, Ltd., India. A PCM stock solution was prepared by dissolving 400 mg of PCM in sterile saline and the volume made up to 20 ml to obtain a final concentration of 20 mg/ml.

### 2.3. Experimental Animals

The experimental protocol was approved by the Institutional Animal Ethics Committee (IAEC), proposal no. JSSCP/IAEC/Pharmacology/05/2017-18. Forty male 12-week-old Wistar rats weighing 120–150 g were obtained and acclimatised in polypropylene cages in standard laboratory conditions (23.4–28.8°C, 54–78% relative humidity) in a 12 h light-dark cycle. They were maintained under standard housing conditions with free excess to a standard diet (M/s. Amruth labs, Bangalore, India) and water* ad libitum* during the experiment.

The animals were randomly divided into five experimental groups of eight rats: control: oral treatment of vehicle 10 ml/kg daily for 14 days; PCM alone: oral treatment of vehicle 10 ml/kg daily for 14 days with intraperitoneal injection of PCM 200 mg/kg daily for 14 days; EL 100: oral treatment of EL 100 mg/kg body weight and intraperitoneal injection of PCM 200 mg/kg 1 hr after the EL treatment daily for 14 days; EL 200: oral treatment of EL 200 mg/kg body weight and intraperitoneal injection of PCM 200mg/kg 1 hr after the EL treatment daily for 14 days; EL 400: oral treatment of EL 400 mg/kg body weight and intraperitoneal injection of PCM 200 mg/kg 1 hr after the EL treatment daily for 14 days.

The body weight of the animals was recorded weekly on day 0, day 7, and day 14 of the treatment. The rats were sacrificed on day 15 after the last treatment followed by overnight fasting. They were anaesthetised with diethyl ether and 2-3 ml of blood samples was collected by cardiac puncture. Serum was obtained for the measurement of protein, albumin, and creatinine levels. The 24 h urine was collected from 11 am on day 14 until 11 am on day 15 for estimation of creatinine clearance levels on day 15. Kidney samples were dissected, trimmed of connective tissues, and washed with normal saline to eliminate blood contamination.

### 2.4. Biochemical Analysis

The samples were allowed to clot and centrifuged at 3000 rpm at 30°c for 15 min and the separated serum was used for the following biochemical estimations using commercially available kits: total protein (Erba Mannheim, Ltd., India), albumin (Precision Biomed Ltd., India), creatinine (Erba Mannheim, Ltd., India), and urea (Erba Mannheim, Ltd., India). The 24 h urine was used for the estimation of creatinine clearance levels (Erba Mannheim, Ltd., India). All assays were performed according to the manufacturers' instructions.

### 2.5. Histopathology of Rat Kidney

After blood sampling, all the animals were sacrificed and subjected to a complete necropsy followed by histopathology. The rat kidneys were identified and carefully dissected out en bloc for histopathological examination. After rinsing in normal saline, sections were taken from each harvested kidney, fixed in 10% formalin, dehydrated in gradual ethanol (50–100%), cleared in xylene, and embedded in paraffin wax. The 5–6 *μ*m sections were prepared using a rotary microtome and stained with haematoxylin and eosin dye for microscopic observation of the histopathological changes.

### 2.6. Statistical Analysis

All the data were expressed as mean ± SD. One-way analysis of variance (ANOVA) was used to determine significant intergroup differences of each parameter. Dunnet's test was used for individual comparisons after significant ANOVA results. A p value <0.05 was considered statistically significant. Graphpad prism 6 software (Graphpad software, Inc. California, USA) was used for the statistical analysis.

## 3. Results

### 3.1. The Effect of EL Treatment on Body Weight

There were no significant changes in the body weight of rats in all treatment groups in comparison to the PCM alone group as shown in Table 1.

### 3.2. Effect on Serum and Urine Biochemistry

Rats from the PCM alone group exhibited significantly decreased (p<0.05) levels of total protein and albumin compared to the control group. In contrast, EL treatment prevented decreases in these parameters, with EL 200 mg/kg and EL 400 mg/kg resulting in nearly normal levels of total protein and albumin (see Table 2). Table 2 shows that serum urea and creatinine were significantly increased (p<0.05) in the PCM alone group compared to the control group. Notably, experimental rats treated with EL 200 mg/kg and EL 400 mg/kg had significantly (p<0.05) lower levels of urea and creatinine compared to PCM alone group. Similar results were observed for creatinine clearance. There was significant decrease in the level of creatinine clearance in the PCM alone group compared to control group. This decrease was prevented in treatment groups, with a significant increase in the creatinine clearance levels in the EL 200 (p<0.01) and EL 400 (p<0.001) groups compared to PCM alone group, suggesting that treatment with the EL extract may protect renal tissue from further damage. Data from BUN/creatinine ratio indicate no significant changes with all the tested groups after treatment.

### 3.3. Effect of EL Treatment on Kidney Histopathology

**Figure 1:**
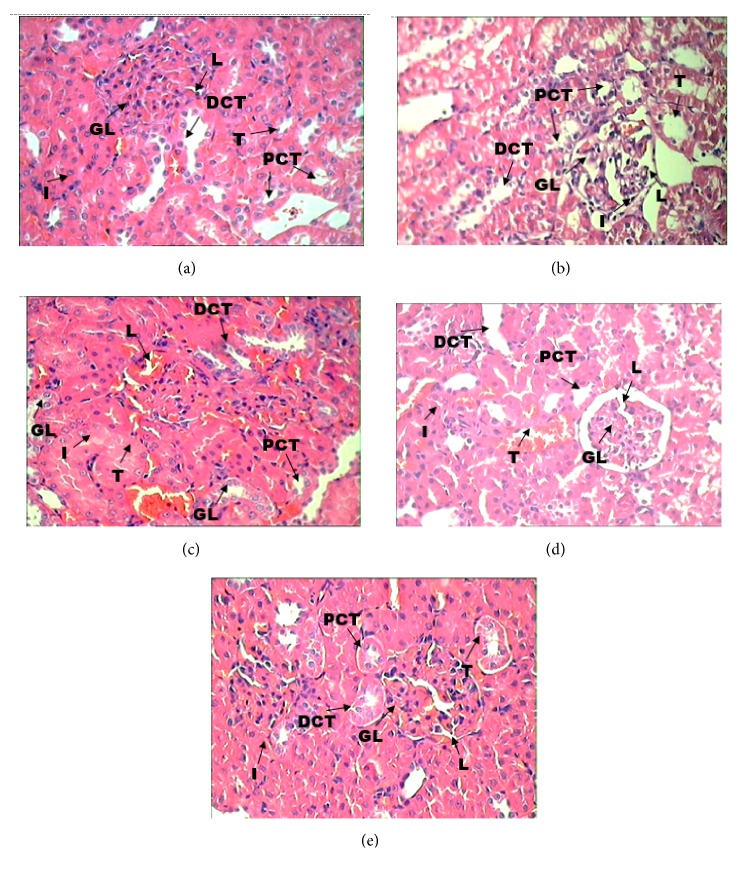
*Histopathology of kidney*. (a) Section of kidney from control group rats showing the normal appearance of glomerulus (GL) and renal tubules (T) including proximal convoluted tubules (PCT) and distal convoluted tubules (DCT) and interstitium (I); (b) sections of the rats treated with PCM alone showed severe tubular degeneration, with wide lumina (L), damaged glomeruli (GL), and interstitial vascular congestion and epithelial degeneration (I); (c) sections of rats treated with EL 100mg/kg showed significant nephroprotection with moderate degenerative changes in the glomeruli (GL) and tubules (T); (d) sections of rats treated with EL 200 mg/kg showed significant nephroprotection with minimal degenerative changes in the glomeruli (GL) and tubules (T)s; (e) sections of rats treated with 400mg/kg showed near normal appearance of glomeruli (GL) and interstitium and tubules (T) including PCT and DCT. H & E stain: 40x.

Histopathological examination of rat kidney sections of PCM alone group showed impaired renal morphology throughout, with severe tubular degeneration, wide lumina, damaged glomeruli, interstitial vascular congestion, and epithelial degeneration. Kidneys from animals concurrently treated with EL 100 mg/kg showed moderate degenerative changes in the glomeruli and tubules, while animals treated with EL 200 mg/kg showed significant nephroprotection with minimal degenerative changes in the glomeruli and tubules. The high dose EL 400 mg/kg provided the highest protection, with near normal appearance of glomeruli, interstitium, and tubules in the kidney, indicating significant nephroprotection (Figure 1).

## 4. Discussion

A clear elevation of urea and creatinine in the PCM alone group provided evidence that the administration of 200 mg/kg of PCM induced kidney injury. Administration of EL at 200 mg/kg and 400 mg/kg concurrently with PCM significantly inhibited the rise in kidney injury markers, i.e., urea and creatinine. Creatinine is produced from the metabolism of protein in muscles, with most creatinine being filtered out of the blood by the kidney and excreted in urine. The glomerular filtration rate (GFR) is a well-known tool to measure the excretory capacity of the kidney. In clinical practice, GFR is obtained from the creatinine clearance in urine samples collected over 24 hours [32]. The significantly higher creatinine clearance rate in the EL 200 (mid dose) and EL 400 (high dose) groups compared to PCM alone group demonstrated the ability of EL to eliminate creatinine from blood into urine, eventually normalising creatinine content in the blood. In renal disease, serum urea accumulates and causes uraemia because the rate of serum urea production exceeds the rate of clearance [29, 33]. The significantly high blood urea in the PCM alone group suggests kidney injury. The administration of EL prevented PCM-induced nephrotoxicity, significantly reducing urea accumulation in the EL 200 (mid dose) and EL 400 (high dose) groups in comparison to the EL 100 (low dose) group.

PCM-induced nephrotoxicity is caused by the toxic effect of N-acetyl-p-benzoquinone imine (NAPQI). PCM is oxidized by cytochrome p-450 and produces reactive intermediate metabolite NAPQI [20, 21]. Another factor in PCM toxicity is formation of reactive oxygen species (ROS) especially superoxide anions. The nephrotoxicity caused by ROS and NAPQI is largely counterpart by glutathione in the early stages of toxicity [34]. However after the depletion of glutathione, NAPQI covalently binds with sulphydryl groups of proteins in later stages of toxicity [31, 34]. The significant decrease (p<0.05) in serum total protein and albumin in the PCM alone group (Table 2) could be due to arylation of protein by NAPQI. Protein content in the blood in EL 200 and EL 400 groups was not significantly decreased compared to the control group, providing evidence that EL is able to minimize the toxic effect of PCM. The biochemical results were also confirmed by the histological findings, which showed preservation of the glomeruli, interstitium, and tubules (Figure 1). Most drugs induce renal injuries that affect the proximal tubes, glomerulus, or more distal parts of the nephron [35]. The intraperitoneal injection of PCM into the PCM alone group caused severe damage to the kidney, with tubular degeneration, wide lumina, damaged glomeruli, interstitial vascular congestion, and epithelial degeneration, whereas EL pretreatment results in significant dose-dependent nephroprotection against PCM-induced nephrotoxicity. Taken together, these results indicate that the EL extract can protect kidneys from the damage caused by PCM and might be a potential therapeutic candidate for PCM-induced nephrotoxicity.

PCM administration can cause substantial peroxidation of membrane lipids and depletion of antioxidants in renal tissue. Various commonly used drugs, such as paracetamol and gentamicin as well as some environmental and industrial toxicants, can cause severe renal damage via the production of highly reactive free radicals [19, 30]. Declining antioxidant status in renal tissue has been shown to partially explain the mechanism of nephrotoxicity induced by PCM as due to free radical production [36]. In addition, an earlier study showed that EL had antioxidant and anti-inflammatory effects [37]. Eurycomanone is a well-known quassinoids of EL and found in water extract of EL which has shown anti-inflammatory activity [38]. Therefore, the protective effect of EL against PCM-induced toxicity could be related to EL's antioxidant and anti-inflammatory activities. Plants containing flavonoids, steroids, and alkaloids possess significant nephroprotective and diuretic activities [13, 39]. Furthermore, the roots of EL contain various alkaloids, which in addition to its antioxidant activity may have contributed to the nephroprotective effects of the EL extract against PCM-induced nephrotoxicity. The nephroprotective effects of EL should be further investigated in other metabolic diseases, such as diabetes and hypertension, which are also associated with kidney damage.

## 5. Conclusion

The present study demonstrated the dose-dependent nephroprotective activity of the EL in a rat model of PCM-induced nephrotoxicity. Pretreatment with EL extract dose-dependently prevented kidney injury as evidenced by serum and urine biochemical analysis and kidney histopathology. In conclusion, EL is a potential nephroprotective agent against drug-induced nephrotoxicity.

## Figures and Tables

**Table 1:** Effect of EL treatment on body weight.

	Body weight (g)				
	Control	PCM alone	EL 100	EL 200	EL 400
Day 0	136.5±12.54	136.5±07.94	145.3±03.91	137.4±09.63	145.0±03.62
Day 7	141.6±19.29	135.8±12.94	138.1±04.73	131.9±17.01	139.6±13.14
Day 14	154.1±23.76	149.3±17.21	146.1±03.87	142.0±16.58	156.0±11.49

Data stated as mean ± SD; ^#^p<0.05 and ^###^p<0.001 in comparison to control; *∗*p<0.05, *∗∗*p<0.01, and *∗∗∗*p<0.001 when compared to PCM alone.

**Table 2:** Effect of EL extract on serum total protein, serum albumin, serum urea, serum creatinine, creatinine clearance levels, and BUN/creatinine ratio.

	Experimental Group				
	Control	PCM alone	EL 100	EL 200	EL 400
serum total protein (g/dl)	11.1±2.0	7.0±1.2^#^	8.9±1.6	9.4±1.4^*∗*^	10.1±1.6^*∗*^
serum albumin (g/dl)	3.6±0.7	1.7±0.7^#^	1.6±0.8	3.1±0.8^*∗*^	3.1±0.3^*∗*^
blood urea (mg/dl)	18.0±1.5	41.7±7.3^#^	42.8±7.5	21.7±3.4^*∗*^	18.0±2.3^*∗*^
serum creatinine (mg/dl)	0.8±0.1	1.6±0.5^#^	1.7±0.3	0.9±0.1^*∗*^	0.8±0.16^*∗*^
creatinine clearance (ml/min)	0.86±0.13^*∗∗∗*^	0.31±0.12^###^	0.30±0.05	0.61±0.22^*∗∗*^	0.87±0.26^*∗∗∗*^
BUN/creatinine ratio	22.8	25.1	24.8	24.4	23.7

Data are mean ± SD; ^#^p<0.05 and ^###^p<0.001 in comparison to control; ^*∗*^p<0.05, ^*∗∗*^p<0.01, and ^*∗∗∗*^p<0.001 when compared to PCM alone.

## Data Availability

The datasets used to support the findings of this study are available from the corresponding author upon request.
