# Corrigendum to “Nephroprotective Effect of Herbal Extract* Eurycoma longifolia* on Paracetamol-Induced Nephrotoxicity in Rats”

**DOI:** 10.1155/2019/9186747

**Published:** 2019-07-21

**Authors:** Sasikala M. Chinnappan, Annie George, Praveen Thaggikuppe Krishnamurthy, YogendraKumar Choudhary, Vandana K. Choudhary, Yesha Ramani, Rashmi Dewangan

**Affiliations:** ^1^Biotropics Malaysia Berhad, Shah Alam, Selangor, Malaysia; ^2^Department of Pharmacology, JSS College of Pharmacy, JSS Academy of Higher Education and Research, Ooty, India; ^3^Etica Clinpharm Pvt. Ltd., Raipur 492001, Chhattisgarh, India

In the article titled “Nephroprotective Effect of Herbal Extract* Eurycoma longifolia* on Paracetamol-Induced Nephrotoxicity in Rats” [1], the affiliation of the third author should be “Department of Pharmacology, JSS College of Pharmacy, JSS Academy of Higher Education and Research, Ooty,” instead of “Etica Clinpharm Pvt. Ltd., Raipur 492001, Chhattisgarh, India.” The corrected affiliations are shown above.
